# Potential steric blockers of interaction between β‐secretase and mutant forms of APP

**DOI:** 10.1002/alz70855_107048

**Published:** 2025-12-25

**Authors:** Mikita Kastsiuchenka, Tatsiana Khrustaleva

**Affiliations:** ^1^ Institute of Physiology of the NAS of Belarus, Minsk, Minsk region, Belarus

## Abstract

**Background:**

Currently, new approaches to the treatment of Alzheimer's disease based on changes in the molecular processes that cause the pathology are becoming more widespread. A promising approach is the development of blockers that, by binding to the proteolysis site on the APP, will prevent the recognition of the site by β‐secretase and sterically interfere with its cleavage. However, mutations of APP have been described that change its propensity for degradation by β‐secretase. Therefore, a promising approach is the search for molecules that tend to block BACE1 binding to both wild‐type APP and its mutant forms.

**Method:**

Peptides with an amino acid chain length of 5‐7 residues were used as potential blockers of the β‐secretase and APP interaction. Their secondary structure was modeled using the PEP‐FOLD 3.5 server. We performed molecular docking of the blocker structures to APP fragments in the Hex 8.0.0 with the shape+electro+DARS correlation type and 5D FFT Mode. We used nTE12 (TEEISEVKMDAE), swTE12 (TEEISEVNLDAE), and seoTE12 (TEEISELKMDAE) fragments, which include the sequences of wild‐type APP, APP with the Swedish mutation, and APP with the Seoul substitution, respectively. We performed docking of substrate‐blocker complexes with the BACE1 molecule (PDB ID: 1xn2) to localize the sites of non‐specific binding.

**Result:**

We investigated 90 structures of potential blockers. Structures with a high affinity to model substrates were selected. Then we excluded substrate‐blocker complexes that bound to the BACE1 active center, since they could limit the access of normal substrates by acting as steric inhibitors of β‐secretase. Guided by these criteria, we selected 10 potential blockers. Peptides KVRKVSV, VTVKRIR, and TIRRIR showed their effectiveness on all model substrates. For LTIRKIR and KIKRVT molecules, the effectiveness was in the case of binding to nTE12 and swTE12. Peptides KVRKLTI, KIRKVS, and KVRKLS showed effectiveness when interacting with nTE12 and seoTE12. Peptides RLRKVTV and RSRVTL are effective for swTE12 and seoTE12. 14 potential blockers showed efficacy against one of the model substrates.

**Conclusion:**

During the work, we selected peptides that had the greatest versatility as blockers to three forms of APP. To confirm their effectiveness, *in vitro* and *in vivo* studies are necessary.

